# Severe malaria in pregnancy in the United States: A case report and management considerations

**DOI:** 10.1016/j.crwh.2026.e00819

**Published:** 2026-05-16

**Authors:** Sebastian Reyes Lizaola, Swati Kumari, Lucia Di Francesco, Julien Chedid, Carolina Cay Martinez

**Affiliations:** Department of Obstetrics and Gynecology, BronxCare Health System, Bronx, NY 10457, USA

**Keywords:** Plasmodium falciparum, Malaria, Congenital malaria, Pregnancy, Preeclampsia, Malaria in pregnancy

## Abstract

Malaria in pregnancy is associated with substantial maternal and fetal morbidity, yet pregnancy-specific guidance remains limited, particularly in non-endemic settings where low clinical suspicion, non-specific symptoms, and testing barriers may delay diagnosis. This report presents a case of severe third-trimester *Plasmodium falciparum* malaria and reviews management considerations.

A nulliparous patient at 36 weeks and 4 days of gestation presented to a U.S. community hospital with fever, headache, and abdominal discomfort, after recent travel to Ghana. Initial evaluation showed maternal fever, fetal tachycardia, thrombocytopenia, and an equivocal peripheral smear. Artemether–lumefantrine was started and escalated to artesunate when repeat testing confirmed *Plasmodium falciparum* with 5.4% parasitemia, meeting criteria for severe malaria. The patient experienced spontaneous preterm prelabor rupture of membranes and worsening thrombocytopenia, prompting transfer to a tertiary center. She underwent induction of labor, delivered vaginally, developed preeclampsia with severe features, and recovered with antimalarial therapy. The newborn remained stable, and congenital malaria was ruled out.

This case highlights the importance of prevention, the diagnostic challenges of malaria in pregnancy, rapid progression to severe disease, and gaps in pregnancy-specific guidance. Algorithms integrating trimester-specific therapy dosing, fetal surveillance, delivery thresholds, and neonatal screening may help standardize care.

## Highlights

• Travel history is fundamental when evaluating fever in pregnancy.
• Repeated malaria testing in pregnancy is essential when clinical suspicion is high.
• Malaria in pregnancy needs guidance on fetal surveillance and delivery timing.

## 1 Introduction

Malaria in pregnancy is a high-risk condition associated with substantial maternal and fetal morbidity [1]. Compared with nonpregnant adults, pregnant individuals are more likely to develop severe disease due to immunologic changes and placental sequestration of parasites [2]. These factors may alter drug metabolism and clinical presentation, reduce early peripheral parasitemia, and contribute to diagnostic delay. Malaria in pregnancy is associated with anemia, preterm birth, fetal growth restriction (FGR), stillbirth, and perinatal mortality [[1], [2]].

Although the World Health Organization (WHO) estimates that millions of pregnancies are exposed to *Plasmodium falciparum* annually [3], pregnancy-specific guidance remains limited regarding trimester-specific drug dosing, fetal monitoring, and delivery thresholds when maternal-fetal status deteriorates. In non-endemic settings, recognition may be delayed because of low prevalence, low clinical suspicion, nonspecific early symptoms, and system-level barriers, including non-optimized testing pathways and overlap with more common conditions [4].

This report describes a case of severe *P. falciparum* malaria in the late third trimester in a non-endemic setting and highlights diagnostic challenges, rapid clinical deterioration, and limitations in pregnancy-specific guidance.

## 2 Case Presentation

A nulliparous patient in her 30s at 36 weeks and 4 days of gestation presented to triage at a community-based hospital in the United States with fever and lower abdominal pain after recent travel to Ghana. Prenatal care had been limited; she initiated care at 29 weeks and had not yet undergone an official ultrasound. Her medical history was notable for sickle cell trait, and she identified as non-Hispanic Black. At the initial prenatal visit, she reported plans for approximately 2 weeks of international travel; however, the destination was not documented, which limited the ability to provide destination-specific counseling; malaria chemoprophylaxis was therefore not initiated.

Earlier on the day of presentation, she had returned for prenatal care and reported recent travel to Ghana. She was discharged after reassuring vital signs and physical examination. During triage evaluation, she reported subjective intermittent fevers for 2 days, a home-recorded temperature of 104 °F (40 °C), severe headache, and abdominal discomfort, without visual symptoms.

On arrival, she was evaluated by a physician. Her temperature was 101.7 °F (38.7 °C), heart rate was 120 beats per minute, and blood pressure was normal. Respiratory and abdominal examinations were benign. Bedside ultrasound demonstrated a biophysical profile of 8/8. Continuous electronic fetal monitoring (EFM) showed a Category II tracing due to fetal tachycardia in the 170 s–180 s, which resolved later, after maternal stabilization.

Initial laboratory testing showed mild anemia (hemoglobin 11.1 g/dL decreasing to 10.5 g/dL) and progressive thrombocytopenia (platelets 110 × 10^9^/L decreasing to 81 × 10^9^/L) on repeat testing after 12 h. Aspartate aminotransferase was normal at 30 unit/L (range 9–36 U/L), alanine aminotransferase was mildly elevated at 45 unit/L (range 5–40 U/L), glucose was 97 mg/dL, and urinalysis showed trace proteinuria. Respiratory infectious panels were negative. Infectious Diseases was consulted and recommended vancomycin, piperacillin/tazobactam, and malaria testing for possible sepsis of unclear source versus malaria.

The initial peripheral smear was reported as “questionable, malaria-like organisms seen.” Infectious Diseases recommended discontinuing broad-spectrum antibiotics and starting artemether-lumefantrine 20 mg/120 mg tablet for a 3-day course; Maternal-Fetal Medicine concurred.

**Fig. 1:**
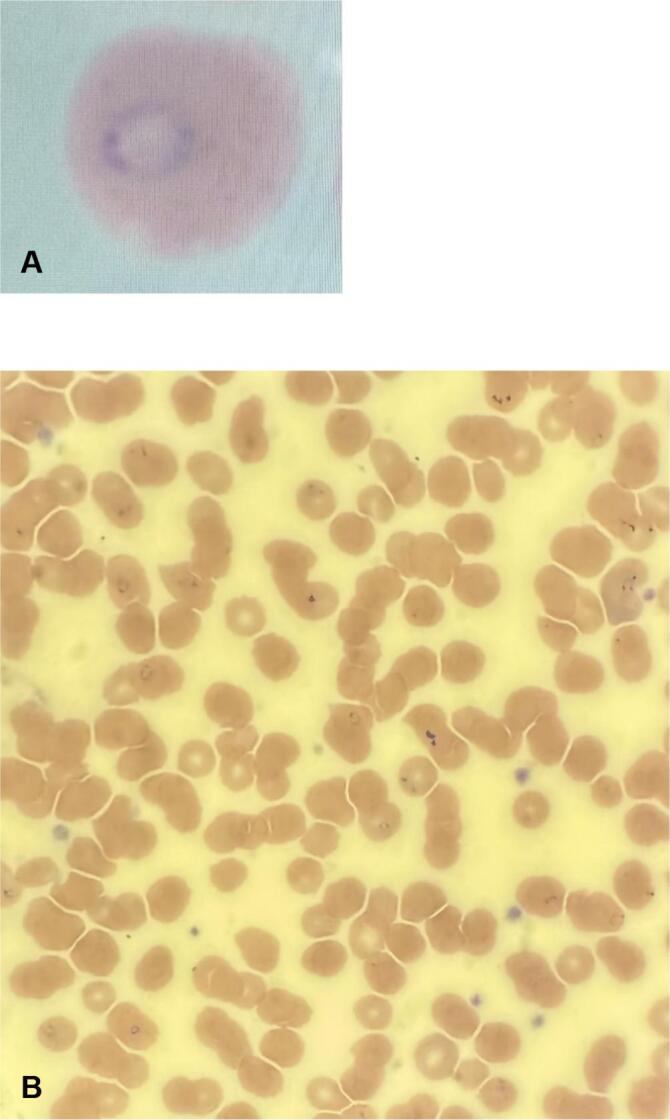
*Plasmodium falciparum* identified on peripheral blood smear by light microscopy. Panels A and B demonstrate parasitized red blood cells under oil immersion at 100× objective magnification. (For interpretation of the references to colour in this figure legend, the reader is referred to the web version of this article.)

Within 24 h, repeat testing confirmed *P. falciparum* infection with 5.4% of red blood cells parasitized (Fig. 1), meeting Centers for Disease Control and Prevention (CDC) criteria for severe malaria. Infectious Diseases recommended discontinuing artemether-lumefantrine and initiating intravenous artesunate at 0, 12, and 24 h, with repeat smear four hours after the third dose. The plan was to transition to oral therapy if parasitemia decreased to 1% or less or continue artesunate with smear monitoring if parasitemia remained above 1%.

Shortly after the first artesunate dose, the patient experienced spontaneous preterm prelabor rupture of membranes at 36 weeks and 5 days. Platelets declined further to 50 × 10^9^/L, and since platelet products were not immediately available, Maternal-Fetal Medicine recommended transfer to a tertiary care center.

After transfer, the patient underwent induction of labor and had an uncomplicated vaginal delivery at 36 weeks and 6 days of a female newborn with Apgar scores of 8 and 9 at 1 and 5 min, respectively, and a birthweight of 2455 g. The patient was diagnosed intrapartum with preeclampsia with severe features due to severe-range blood pressures, completed magnesium sulfate therapy, and was discharged on oral labetalol. As parasitemia improved, she was transitioned to oral antimalarial therapy and recovered without platelet transfusion. The infant remained stable and congenital malaria was ruled out by a peripheral-blood parasite smear. The remaining postpartum course was uneventful.

## 3 Discussion

This case highlights diagnostic difficulty in a non-endemic setting, rapid progression to severe disease in late gestation, and limited pregnancy-specific management guidance.

Imported malaria in pregnancy remains uncommon in the United States, but cases have increased with global travel [5]. In 2023, 10 locally acquired malaria cases were also reported in Arkansas, Florida, Maryland and Texas, caused by *P. vivax* and *P. falciparum* [6]. A retrospective study of pregnancy-related hospitalizations from 2002 to 2017 identified 1761 malaria-associated admissions, with an increasing trend over time [5].

These hospitalizations occurred disproportionately among patients identifying as non-Hispanic Black and were associated with increased odds of severe preeclampsia, preterm labor, and stillbirth [5]. Consistent with these reported associations, the patient identified as non-Hispanic Black, developed preeclampsia with severe features and had a preterm delivery.

Malaria during pregnancy is associated with adverse maternal and neonatal outcomes. A recent meta-analysis reported increased odds of anemia (OR 2.40; 95% CI 1.87–3.06), low birthweight (OR 1.99; 95% CI 1.60–2.48), preterm birth (OR 1.65; 95% CI 1.29–2.10), and stillbirth (OR 1.40; 95% CI 1.15–1.71) [7]. Disease is often more severe in the first two pregnancies because subsequent pregnancies are associated with antibodies that inhibit placental parasite sequestration [8]. Studies have associated *P. falciparum* infection with preeclampsia or eclampsia in primiparous pregnancies and gestational hypertension in multiparous individuals [9].

Published case reports show variable presentations and outcomes, including a U.S. case of maternal sepsis at 31 weeks, followed by treatment and subsequent term delivery [8], and a report from Colombia of *P. vivax* at 37 weeks requiring cesarean delivery due to clinical deterioration, anemia, preeclampsia with severe features and thrombocytopenia requiring red blood cell and platelet transfusion [10].

Professional organizations provide general guidance for malaria in pregnancy. The American College of Obstetricians and Gynecologists (ACOG) acknowledges its detrimental effects [11], recommends avoiding travel to areas at risk, and endorses chemoprophylaxis when travel is unavoidable [12]. This case highlights the importance of obtaining a detailed travel history to support destination-specific counseling and chemoprophylaxis when indicated. The Royal College of Obstetricians and Gynecologists (RCOG) notes that malaria may present with non-specific flu-like symptoms even up to a year after returning from an endemic region [13].

The WHO defines severe malaria by clinical manifestations and laboratory criteria (Table 1) [14], whereas the CDC criteria also include parasitemia of 5% or greater, hemoglobin less than 7 g/dL, and disseminated intravascular coagulation (DIC) [15].

**Table 1:** Diagnostic clinical manifestations and laboratory criteria for severe malaria according to the World Health Organization (WHO) guideline [14].

World Health Organization (WHO) characteristics of severe malaria
**Clinical manifestations:** • Impaired consciousness, prostration, multiple convulsions, respiratory distress, jaundice, pulmonary edema, shock. **Laboratory findings** • Acidosis (base deficit of more than 8 mEq/L, plasma bicarbonate of less than 15 mmol/L, venous plasma lactate more than or equal to 5 mmol/L), hypoglycemia (blood glucose less than 2.2 mmol/L or less than 40 mg/dL), renal impairment (serum or plasma creatinine more than 265 μmol/L or 3 mg/dL, or blood urea >20 mmol/L), hyperparasitemia (more than 10% of red blood cells parasitized by *Plasmodium falciparum*)

The patient initially presented with nonspecific symptoms, and earlier evaluation on the day of admission was not diagnostic. In pregnant patients, peripheral parasitemia may be low or intermittently detectable, causing false-negative or equivocal smears early in disease [16]. The WHO recommends repeat testing every 12–24 h for up to 3 sets when suspicion remains high despite an initial negative smear [17]. In this case, the first smear was equivocal, whereas repeat testing within 24 h showed parasitemia greater than 5%.

Once confirmed, management aligned with recommendations for intravenous artesunate, first-line treatment for severe malaria [[14], [15]]. Artemisinin-based combination therapies, including artemether-lumefantrine, are options in the second and third trimesters for uncomplicated infection or follow-on therapy after artesunate [18].

Important pregnancy-specific questions remain unanswered. Pregnancy alters antimalarial pharmacokinetics, and reduced exposure to some agents may theoretically decrease efficacy [[8], [19]]. Guidelines provide limited direction on delivery timing, and fetal surveillance when maternal status worsens in late gestation [[14], [15]].

In this case, fetal tachycardia improved after maternal stabilization, but worsening thrombocytopenia raised unresolved clinical questions. The RCOG states that uncomplicated malaria alone is not an indication for induction [13]; however, recommendations for complex late-pregnancy presentations are limited. Regarding EFM, the RCOG also notes that it may show fetal tachycardia, bradycardia or late fetal decelerations, particularly with maternal fever [13].

Although malaria increases the risk of FGR, stillbirth, and neonatal malaria, standardized protocols for fetal surveillance and congenital malaria screening are lacking [7]. The RCOG recommends fetal growth surveillance after diagnosis and treatment [13]. Protocolized newborn screening after antepartum malaria may identify congenital malaria early, permit timely treatment, and reduce long-term complications.

## 4 Conclusion

This case reinforces the need for pregnancy-specific malaria algorithms. It illustrates diagnostic challenges unique to pregnancy, including equivocal smears and rapid progression to severe disease, and highlights the importance of prevention, early diagnosis in non-endemic settings, and standardized congenital malaria screening. Prospective research and consensus-driven protocols may help standardize care and provide guidance on trimester-specific dosing, maternal and fetal monitoring, and delivery thresholds for pregnant patients with malaria [20].

## Contributors

Sebastian Reyes Lizaola contributed to patient care, conception of the case report, acquiring and interpreting the data, drafting the manuscript and undertaking the literature review.

Swati Kumari contributed to interpreting the data, drafting the manuscript and undertaking the literature review.

Lucia Di Francesco contributed to patient care, conception of the case report and drafting the manuscript.

Julien Chedid contributed to patient care, acquisition of data and revising the article critically for important intellectual content.

Carolina Cay Martinez contributed to patient care, conception of the case report and revising the article critically for important intellectual content.

All authors approved the final submitted manuscript.

## Patient consent

Written informed consent was obtained from the patient for publication of the case report and accompanying images.

## Provenance and peer review

This article was not commissioned and was peer reviewed.

## Declaration of generative AI and AI-assisted technologies in the writing process

During the preparation of this work the authors used ChatGPT (OpenAI, GPT-5.3) to support grammar and wording refinement, to improve the clarity and readability of the manuscript. AI was not used to interpret clinical data, perform analysis, or draw any type of scientific conclusions. After using ChatGPT, the authors reviewed and edited the content as needed and take full responsibility for the content of the published article.

## Funding

This work did not receive any specific grant from funding agencies in the public, commercial, or not-for-profit sectors.

## Declaration of competing interest

The authors declare that they have no competing interest regarding the publication of this case report.
